# 3-Amino-1,2,4-Triazole Induces Quick and Strong Fat Loss in Mice with High Fat-Induced Metabolic Syndrome

**DOI:** 10.1155/2020/3025361

**Published:** 2020-04-13

**Authors:** Valéria Nunes-Souza, Nelson Miguel Dias-Júnior, Marcos Antônio Eleutério-Silva, Vanessa P. Ferreira-Neves, Fabiana Andréa Moura, Natalia Alenina, Michael Bader, Luíza A. Rabelo

**Affiliations:** ^1^Laboratório de Reatividade Cardiovascular, Setor de Fisiologia, Instituto de Ciências Biológicas e da Saúde, Universidade Federal de Alagoas, Maceió, Alagoas, Brazil; ^2^Núcleo de Síndrome Metabólica, Universidade Federal de Alagoas, Maceió, Alagoas, Brazil; ^3^Max-Delbrück-Center for Molecular Medicine, Berlin, Germany; ^4^Departamento de Fisiologia e Farmacologia, Centro de Biociências (CB), Universidade Federal de Pernambuco (UFPE), Recife, Pernambuco, Brazil; ^5^Faculdade de Medicina, Universidade Federal de Alagoas, Maceió, Alagoas, Brazil; ^6^Faculdade de Nutrição (FANUT), Universidade Federal de Alagoas, Maceió, Alagoas, Brazil; ^7^German Center for Cardiovascular Research (DZHK), Partner Site Berlin, Germany; ^8^Berlin Institute of Health (BIH), Berlin, Germany; ^9^Charité University Medicine, Berlin, Germany; ^10^Institute for Biology, University of Lübeck, Germany

## Abstract

**Background:**

Obesity is a growing epidemic with limited effective treatments and an important risk factor for several diseases such as metabolic syndrome (MetS). In this study, we aimed to investigate the effect of 3-amino-1,2,4-triazole (ATZ), an inhibitor of catalase and heme synthesis, in a murine model for MetS.

**Methods:**

Male C57BL/6 mice with high-fat diet-induced MetS received ATZ (500 mg·kg^−1^·24 h^−1^) for 12 weeks.

**Results:**

The HFD group showed increased blood pressure and body weight, enhanced fat deposition accompanied by an increase in adipocyte diameter, and decreased lipolysis in white adipose tissue (WAT). The expression of genes related to inflammation was increased in WAT of the HFD group. Concurrently, these mice exhibited an increase in leptin, nonesterified fatty acid (NEFA), insulin, and glucose in plasma, coupled with glucose intolerance and insulin resistance. Strikingly, ATZ prevented the increase in blood pressure and the HFD-induced obesity as observed by lower body weight, WAT index, triglycerides, NEFA, and leptin in plasma. ATZ treatment also prevented the HFD-induced increase in adipocyte diameter and even induced marked atrophy and the accumulation of macrophages in this tissue. ATZ treatment also improved glucose metabolism by increasing glucose tolerance and insulin sensitivity, GLUT4 mRNA expression in WAT in parallel to decreased insulin levels.

**Conclusions:**

In the context of HFD-induced obesity and metabolic syndrome, the fat loss induced by ATZ is probably due to heme synthesis inhibition, which blocks adipogenesis by probably decreased RevErb*α* activity, leading to apoptosis of adipocytes and the recruitment of macrophages. As a consequence of fat loss, ATZ elicits a beneficial systemic antiobesity effect and improves the metabolic status.

## 1. Introduction

Obesity is a growing epidemic with limited effective treatments and an important risk factor for several diseases such as the metabolic syndrome (MetS). This condition is characterized by a group of symptoms [1], including obesity, hypertension, hypertriglyceridemia, dyslipidemia, glucose intolerance, and insulin resistance [1, 2]. White adipose tissue (WAT), the primary lipid storage depot [3], is central to the development of the MetS, while being a powerful endocrine organ [4]. Increased fat accumulation is closely related to cardiometabolic diseases, and thereby, the control of fat deposition becomes pivotal to maintain a healthy life [3]. Currently, one of the most discussed theories that attempts to explain the pathological conditions associated with MetS involves the participation of oxidative stress, resulting from changes in redox state, with a predominance of prooxidant systems over antioxidants [5].

3-Amino-1,2,4-triazole (ATZ) is a heterocyclic organic compound widely used as a catalase inhibitor [6], a ubiquitous enzyme which metabolizes H_2_O_2_ to oxygen and water. Probably linked to its main function, this compound also inhibits *α*-oxidation [7], fatty acid synthesis, and lipogenesis in isolated hepatocytes. In human differentiated adipocytes, ATZ treatment impaired the antioxidant defense system and induced inflammation [8]. Park et al. [9] have demonstrated that the pharmacological or genetic inhibition of catalase alters macrophage activation and thereby induces inflammation of adipose tissue, suggesting a novel role of endogenous catalase in macrophage polarization in adipose tissue. In animals, the median lethal dose (LD_50_), which ensures low acute toxicity [10]. However, the doses vary according to some species already studied: in mice, the LD_50_ was 11,000 mg · kg^−1^; in sheep, 4,000 mg · kg^−1^ was fatal; in rats, no signs of toxicity with 4,080 mg · kg^−1^ were observed [11]; in bacterial and cultured mammalian cells and rodents exposed in vivo, the ATZ was not genotoxic [12]. Steinhoff and coauthors did not observe carcinogenic activity of ATZ in golden hamsters or in mice fed with ATZ in a lifespan test at dietary levels of 1, 10, and 100 ppm (rg amitrole · g^−1^ food), until they died spontaneously. However, in rats, thyroid and pituitary gland tumors were detected, induced by ATZ [13].

ATZ also inhibits aminolevulinic acid dehydratase, a key enzyme in heme synthesis. Heme activates the transcription repressor RevErb*α*, which is essential for adipocyte differentiation [14]. Thus, ATZ may inhibit adipogenesis by blocking the synthesis of heme. It has been shown that ATZ induces fat loss and decreases plasma triacylglycerol levels in mice [15]. However, the relevance of this phenomenon in MetS, as well as the mechanism by which ATZ induces fat loss, still remain unclear. We have inquired if ATZ decreases lipid storage by increasing inflammation and cell death, by decreasing adipogenesis, and/or by lipolysis. To address these questions, we used high-fat diet- (HFD-) induced MetS in mice, a widely used model to test pharmacological effects on obesity [16].

## 2. Methods

### 2.1. Ethics Statement and Animal Care

All experiments reported here have been conducted in accordance with the National Institutes of Health Guide for the Care and Use of Laboratory Animals (Institute of Laboratory Animal Resources, National Academy Press, Washington, DC, 1996). The procedures were approved by the Ethical Committee of the Federal University of Alagoas (029/2014). All the animals were housed in an animal facility on a 12-hour light/dark cycle, and food and water were available *ad libitum*.

### 2.2. Diets and Research Design

C57BL/6 male mice (4-6 weeks old) were randomly divided into three groups: a control (CT, *n* = 7), which was fed with standard diet (caloric intake = 11.8% fat), the second group was fed with HFD (caloric intake = 58.4% fat, primarily lard) (HFD, *n* = 6), and the third group was fed with HFD treated by dietary supplementation with ATZ (HFD+ATZ, *n* = 8; 500 mg·kg^−1^ 24 h^−1^). The HFD was prepared according to Nunes-Souza et al. [16], and all components were purchased from Rhoster® LTDA (São Paulo, Brazil) and Sigma® (Seelze, Germany). The animals were evaluated during 20 weeks in total. However, the treatment with ATZ (Sigma®, Seelze, Germany) started at the beginning of the eighth week of HFD feeding. The dose and mode of application of ATZ administration were determined in a pilot experiment, which indicated that 500 mg·kg^−1^ of ATZ promotes lipolytic effect and reduction of visceral adiposity (unpublished data). The dose of ATZ was adjusted weekly, taking into account the mean body weight and the food intake, which were assessed weekly in a semianalytical scale.

### 2.3. In Vivo and In Vitro Experiments

#### 2.3.1. Systolic Blood Pressure and Metabolic Assessments

At the end of the treatment, the animals were adapted to a small mouse holder during one week. The measurement of systolic blood pressure was recorded by tail plethysmography (PowerLab®, ADInstruments, Melbourne, Australia).

Intraperitoneal (i.p) glucose tolerance test (GTT) was carried out in overnight-fasted mice (12 hours), and insulin tolerance test (ITT) was performed in overnight fed; both were conducted accordingly as described previously [16]. The product of fasting triglyceride and glucose levels (TyG index), a validated and highly sensitive marker of insulin resistance, was calculated using the following formula [17, 18]: TyG index = Ln [triglyceride (mg · dL^−1^) × glucose (mg · dL^−1^)/2].

#### 2.3.2. Lipolysis

*(1) In Vivo*. In an independent group of animals in fed conditions, the lipolysis *in vivo* was performed by administration of 1 mg·kg^−1^, i.p selective adrenergic *β*3-receptor agonist, the CL-316,243 hydrate (C5976; Sigma-Aldrich®, Seelze, Germany) [19, 20]. The blood was collected from tail vein before the administration and after, in 15 and 30 minutes. Nonesterified fatty acid (NEFA) was measured in plasma and normalized by the white adipose tissue (WAT) index, which was obtained after euthanasia.

*(2) In Vitro*. To determine the influence of ATZ (50 mmmol·L^−1^), catalase (1,200 mL^−1^), and H_2_O_2_ (0.1 mmmol·L^−1^) separately, we performed the lipolysis *in vitro* in WAT collected from C57Bl/6 mice feeding chow diet. The tissue was incubated in a medium of culture (DMEM, Gibco® 11880; Darmstadt, Germany) in a bath (37°C; 95% O_2_; 5% CO_2_) for 30 minutes for collection of basal time (time 0). Immediately after that, the medium was imbibed with CL-316,243 0.1 mM alone and in combination with ATZ, CAT, and H_2_O_2_ separately. The NEFA were measured in the medium in 0, 90, and 180 minutes of incubation and normalized by the amount of fat used for stimulation.

### 2.4. Euthanasia and *Ex Vivo* Experiments

In fasted state, all animals were anesthetized (100 mg·kg^−1^ ketamine, 10 mg·kg^−1^xylazine, i.p). In the sequence, animals were euthanized by exsanguination through the right ventricle puncture. Plasma was obtained after blood centrifugation (2,150 g) at 4°C for 10 minutes. The epididymal and perirenal WAT, as well as interscapular brown adipose tissue (BAT), were removed, weighed, and stored at −80°C until further analysis. WAT index was calculated using the following formula: WAT index (%) = [(epididymal fat_(g)_ + perirenal fat_(g)_)/(body weight_(g)_)∗100]. The relative weight (%) of tissue was calculated using the following formula: relative weight (%) = [(tissue_(g)_/body weight_(g)_)∗100]. The right tibias were removed, and the length was measured using a pachymeter.

#### 2.4.1. Circulating Biochemical Analysis

Fasting total cholesterol (TCOL), triglycerides (TG) (Labtest®, Lagoa Santa, Brazil), and NEFA (Wako Chemicals GmbH®, Germany) levels in plasma were assayed using commercial kits following the manufacturers' instructions and performed in a microplate (Thermo Scientific®, Software 2.4 Multiskan Spectrum, Finland). ELISA assays were used to measure insulin and leptin levels (Millipore®, Schwalbach, Germany) according to the manufacturers' instructions.

#### 2.4.2. Evaluation of eWAT Redox Status

A piece of frozen eWAT was homogenized in a RIPA lysis buffer (pH 7.5; Cell Signaling Technology®, Beverly, MA) containing protease and phosphatase inhibitor cocktails (Roche®, Mannheim, Germany). Total protein levels were determined by the Bradford assay [21]. The eWAT catalase activity was measured according to Xu and colleagues [22], and enzyme activity was expressed in *μ*mol·min·mL^−1^ per eWAT protein (mg·mL^−1^) [21]. Total superoxide dismutase (SOD) activity was assessed with a commercial colorimetric kit (#19160, Sigma®, Seelze, Germany) following the manufacturer's instructions. Lipid peroxidation in eWAT was determined by measuring the thiobarbituric acid reactive substances (TBARS), as a marker of oxidative stress, mainly malondialdehyde (MDA). The quantification was performed according to Ohkawa et al. [23] with modifications, as previously described [24]. Data were normalized per total protein concentration, measured by Bradford [21] and expressed as nM·mg protein^−1^.

Hydrogen peroxide (H_2_O_2_) was measured by fluorescence using the Amplex® UltraRed hydrogen peroxide (10-acetyl-3,7-dihydroxiphenoxazine) assay (Invitrogen®, Paisley, United Kingdom) as described [24].

#### 2.4.3. Gene Expression in Epididymal White Adipose Tissue

Gene expression was determined by real-time quantitative polymerase chain reaction (qPCR). Briefly, total RNA was isolated from epididymal white adipose tissue (eWAT) by using trizol (TRizol® Reagent, Darmstadt, Germany), quantified by spectrophotometry, and 1 *μ*g was used for the synthesis of cDNA by reverse transcriptase (Invitrogen®). Subsequently, the product was amplified using the GoTaq qPCR Master Mix (Promega®; Mannheim, Germany) by real-time quantitative PCR (ABI 7900HT Real-Time PCR System-Applied Biosystems®, Darmstadt, Germany). mRNA was quantified as a relative value compared with an internal reference, GAPDH. Quantitative values for mRNA expression were obtained by the parameter 2^−*ΔΔ*Ct^ method [25]. The primers used for real-time quantitative PCR are listed in Table 1.

#### 2.4.4. Histological Analysis in eWAT

Small fragments of eWAT was fixed in 4% buffered formaldehyde, embedded in paraffin, and sectioned at 10 *μ*m. H&E images were used for the determination of mean adipocyte size and macrophage count. The sections were observed under an Olympus BX51 attached DP70 Digital Camera System (Tokyo, Japan); the fields were evaluated with final magnification of 20x (50 *μ*m). Digital photographs were taken from each section, adipocyte size expression were quantified using the “ImageJ” image processing software (NIH, Bethesda, MD, USA), and the macrophage count was performed on the average of 4 fields per animal (macrophage/field) [26–28].

### 2.5. Data Analysis

Results are expressed as mean ± SEM, and “*n*” indicates the number of animals used in the experiment. The dose-response curves of the different groups were compared by two-way ANOVA followed by Bonferroni's correction. One-way ANOVA was used for other comparisons followed by Bonferroni tests using GraphPad Prism® version 5.0 for Windows. A value of *p* < 0.05 was considered significant.

## 3. Results

### 3.1. ATZ Decreases Body Weight, WAT Depots, Lipid Profile, and Blood Pressure in High Fat-Induced MetS Mice

**Figure 1:**
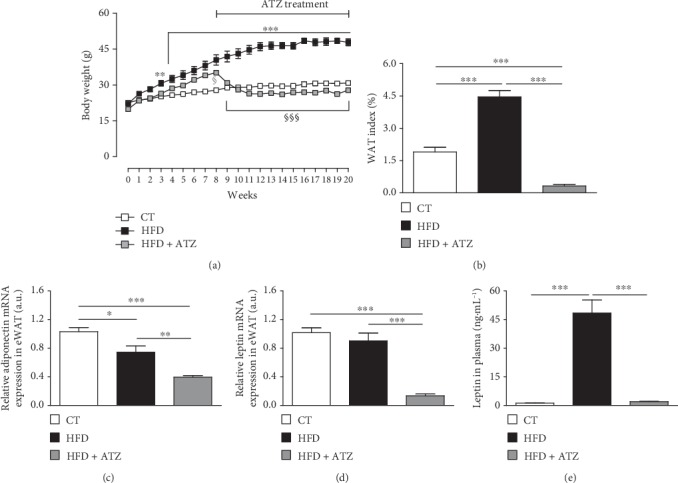
(a) Body weight during the weeks of treatment (g); (b) WAT index (%); (c, d) relative gene expression of adiponectin and leptin, respectively, in epididymal white adipose tissue (eWAT) (arbitrary units); (e) leptin levels in plasma (ng·mL^−1^) of C57BL/6 mice after dietetic intervention (chow and high-fat) and oral treatment with ATZ (500 mg·kg^−1^). Each point on the graph represents the mean ± SEM. ANOVA (two-way): ^∗∗^*p* < 0.001; ^∗∗∗^*p* < 0.001 HFD *vs*. CT; ^§^*p* < 0.05; ^§§§^*p* < 0.001 HFD+ATZ *vs.* HFD. Each bar graph represents the mean ± SEM. ANOVA (one-way): ^∗^*p* < 0.05, ^∗∗^*p* < 0.01, and ^∗∗∗^*p* < 0.001.

MetS was induced in mice by HFD for 20 weeks. At week 8, one group was additionally treated with 500 mg · kg^−1^ 24 h^−1^ of ATZ. The HFD group showed a significant increase in body weight from the third until the 20^th^ week of HFD consumption compared to the control (Figure 1(a)). Strikingly, after one week of ATZ treatment until the end of the experimental protocol (from the 9^th^ week until 20^th^ week), the body weight decreased significantly compared to the HFD group (Figure 1(a) and Table 2). The data demonstrate the effect of HFD on body weight gain, as well as the effect of ATZ on weight loss. Due to the higher caloric and lipid content of HFD, food intake was reduced in both groups fed a HFD irrespective of ATZ treatment. Tibia length and total plasma protein were similar between the groups (Table 2).

After HFD consumption, the systolic blood pressure, WAT index, and epididymal and perirenal adipose tissue increased significantly compared to the control (CT) group. However, after ATZ treatment, all of these parameters were significantly reduced (Figure 1(b) and Table 2). On the other hand, brown adipose tissue significantly increased only in the HFD+ATZ group (Table 2), suggesting a probable action of ATZ treatment in adipocyte differentiation and thermogenesis.

The expression of mRNA for adiponectin in WAT was significantly decreased in both, the HFD and HFD+ATZ groups compared to CT (Figure 1(c)). Consistently with the increased amount of visceral WAT and an unchanged mRNA level (Figure 1(d)), the plasma levels of leptin were significantly higher in HFD animals (Figure 1(e)). Interestingly, in HFD+ATZ mice, both protein and gene expressions of leptin were significantly lower compared to the HFD group (Figures 1(d) and 1(e), respectively).

The fasting plasmatic levels of TCOL and nonesterified fatty acid (NEFA) were significantly increased in the HFD group compared to CT, with no changes in TG levels (Table 2). Concurrent with the decrease in fat depots, the levels of TG and NEFA, but not TCOL, were also significantly decreased in the HFD+ATZ group (Table 2).

**Figure 2:**
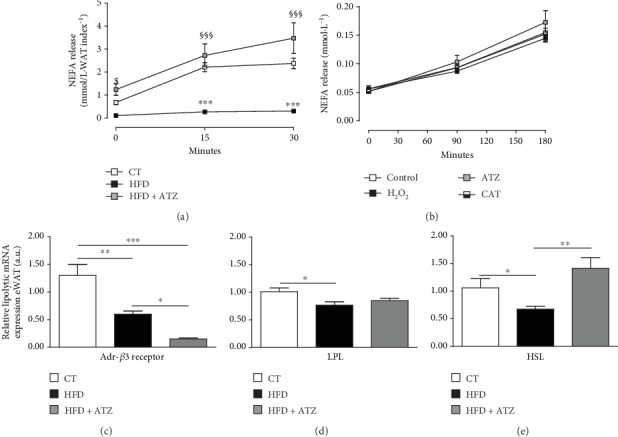
(a, b) NEFA release after lipolysis *in vivo* (mmol.L^−1^·WAT index^−1^) and *in vitro* (mmol.L^−1^), respectively; (c–e) relative gene expression of Adr*β*3, LPL, and HSL in eWAT (arbitrary units, respectively) of C57BL/6 mice after dietetic intervention (chow and high-fat) and oral treatment with ATZ (500 mg·kg^−1^). Each point on the graph represents the mean ± SEM. ANOVA (two-way): ^∗∗∗^*p* < 0.001 HFD *vs.* CT; ^§^*p* < 0.05; ^§§§^*p* < 0.001 HFD+ATZ *vs.* HFD. Each bar graph represents the mean ± SEM. ANOVA (one-way): ^∗^*p* < 0.05, ^∗∗^*p* < 0.01, and ^∗∗∗^*p* < 0.001.

The lipolytic activity of WAT was measured *in vivo* by stimulation with CL-316,243 hydrate, a selective *β*3-adrenoceptor agonist. The release of NEFA was significantly lower over time (15 and 30 minutes after stimulation) in the HFD group compared to the CT group (Figure 2(a)). In contrast, NEFA release was significantly higher before and 15 and 30 minutes after stimulation in the HFD+ATZ group than in the HFD group and indistinguishable from controls, which could explain the lower fat depot in these animals. The data demonstrate the decrease in lipolysis induced by HFD, as well as the increase in this parameter induced by ATZ. The basal NEFA at time point 0 was different among the three groups because it was normalized by the WAT index (mmol/L·WAT index^−1^), which is different among groups.

In order to determine the influence of ATZ, catalase, and H_2_O_2_ separately, the same lipolysis assay was performed *in vitro* in eWAT. The results showed no significant difference at baseline and after 90 and 180 minutes of stimulation between the groups that received only CL-316,243 hydrate or associated with ATZ, catalase, or H_2_O_2_ (Figure 2(b)), suggesting that the elevated lipolysis induced by ATZ *in vivo* does not involve the direct action of these substances on the breakdown of triglycerides within adipocytes.

Analysis of the expression of genes involved in lipolysis showed that HFD induced a significant decrease in adrenergic *β*3-receptor (*β*3), lipoprotein lipase (LPL), and hormone-sensitive lipase (HSL) mRNA in WAT (Figures 2(c)–2(e)). ATZ treatment decreased even further the levels of *β*3 mRNA but did not change LPL mRNA expression compared to the HFD group (Figures 2(c) and 2(d)). However, HSL mRNA expression was significantly higher in the HFD+ATZ animals than in the HFD group (Figure 2(e)), which could explain the increased lipolysis induced by ATZ *in vivo*, since HSL is a lipolytic enzyme responsible for the breakdown of triglycerides stored in adipocytes to free fatty acids.

**Figure 3:**
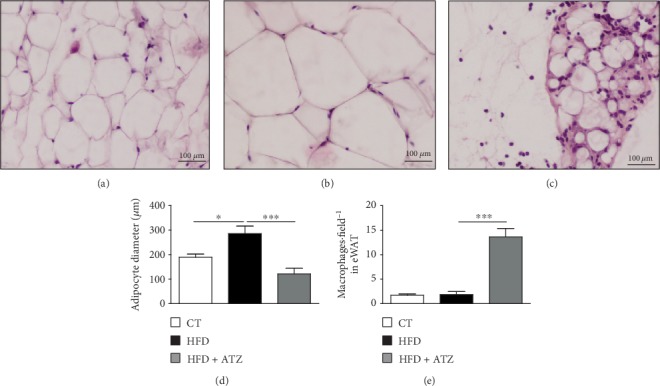
Photomicrographs of a section of the epididymal adipose tissue show the adipocytes after staining with hematoxylin-eosin (HE; (a) CT, (b) HFD, and (c) HFD+ATZ); (b) adipocyte with triglyceride accumulation confirmed by the larger diameter of the cell (*vs.* CT group); (c) several nuclei probably of infiltrating macrophages; (d) white adipocyte diameter (*μ*m) in eWAT; (e) macrophage/field in WAT. Each bar graph represents the mean ± SEM. ANOVA (one-way): ^∗^*p* < 0.05; ^∗∗∗^*p* < 0.001.

Histological analysis of eWAT showed a significant increase in the adipocyte diameter after HFD consumption, indicating large accumulation of triglycerides. ATZ prevented this effect and even induced marked atrophy and an accumulation of macrophages in this tissue (Figures 3(a)–3(e)).

### 3.2. ATZ Modulates Markers of Inflammation, Cell Death, Adipogenesis, and Oxidative Status in eWAT

**Figure 4:**
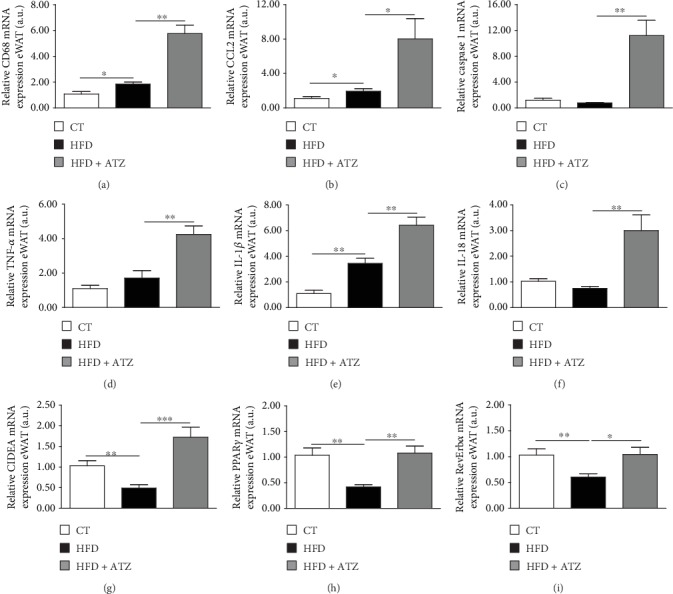
Relative gene expression of (a) CD68, (b) CCL2, (c) caspase 1, (d) TNF-*α*, (e) interleukin-1*β*, (f) interleukin 18, (g) CIDEA, (h) PPAR*γ*, and (i) RevErb*α* in eWAT (arbitrary units) of C57BL/6 mice after dietetic intervention (chow and high-fat) and oral treatment with ATZ (500 mg·kg^−1^). Each bar graph represents the mean ± SEM. ANOVA (one-way): ^∗^*p* < 0.05, ^∗∗^*p* < 0.01, and ^∗∗∗^*p* < 0.001.

We next hypothesized that the marked atrophy in eWAT after ATZ treatment could be due to the activation of pathways involved in inflammation, cell death, inhibition of adipogenesis, and/or high oxidative stress. Related to the inflammatory pathway, the gene expression of cluster of differentiation 68 (CD68), monocyte chemoattractant protein 1 (MCP1 or CCL2), and interleukin-1*β* (IL-1*β*) was significantly increased in the HFD group compared to the CT group, and the ATZ treatment increased it even more (Figures 4(a), 4(b), and 4(e), respectively). The amount of mRNA for caspase 1, tumor necrosis factor alpha (TNF-*α*), and IL-18 was not different between CT and HFD, but ATZ treatment significantly increased their levels compared to HFD (Figures 4(c), 4(d), and 4(f), respectively). The mRNA for CIDEA, a cell death marker, and also for PPAR*γ* (peroxisome proliferator-activated receptor gamma) and RevErb*α*, both involved in adipogenesis, were significantly decreased in the HFD compared to the CT group. However, in the ATZ group, the expression of these genes increased significantly compared to the HFD group (Figures 4(g)–4(i), respectively).

**Figure 5:**
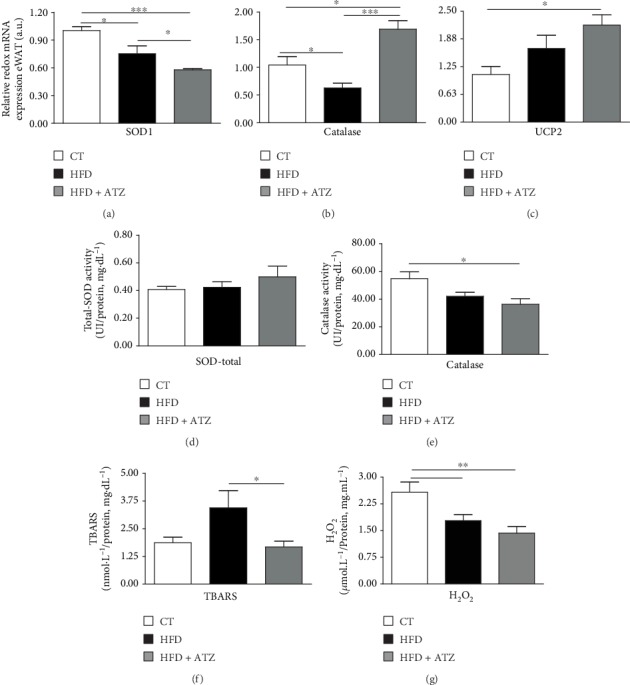
(a–c) Relative gene expression of SOD1, catalase, and UCP2 in WAT (arbitrary units), respectively; (d) SOD activity (UI/protein, mg·dL^−1^) in eWAT. (e) Catalase activity (UI/protein, mg·dL^−1^) in eWAT; (f) TBARS levels (nmol·L^−1^/protein, mg·dL^−1^) in WAT; (g) H_2_O_2_ levels (*μ*mol·L^−1^/protein, mg·mL^−1^) in eWAT of C57BL/6 mice after dietetic intervention (chow and high-fat) and oral treatment with ATZ (500 mg·kg^−1^). Each bar graph represents the mean ± SEM. ANOVA (one-way): ^∗^*p* < 0.05, ^∗∗^*p* < 0.01, and ^∗∗∗^*p* < 0.001.

In the evaluation of the redox profile, mRNA for SOD1 (the Cu-Zn-cytoplasmic isoform) and catalase were significantly decreased in the HFD compared to the CT group (Figures 5(a) and 5(b)). However, the gene expression of mitochondrial uncoupling protein 2 (UCP2), an anion carrier protein, showed no significant differences between the HFD group and the CT group (Figure 5(c)). ATZ treatment significantly further decreased SOD expression in eWAT. Interestingly, catalase and UCP2 mRNA expression were increased even compared to the control group (Figures 5(b) and 5(c)). Despite changes in its mRNA, total SOD activity in eWAT was not different between the groups (Figure 5(d)). Catalase activity was slightly decreased after ATZ treatment despite upregulation of its mRNA (Figure 5(e)). Lipid peroxidation, measured by TBARS levels in eWAT, was decreased by ATZ treatment in the HFD groups (Figure 5(f)). On the other hand, the levels of H_2_O_2_ in eWAT were equally decreased in the HFD and HFD+ATZ groups compared to the CT group (Figure 5(g)).

### 3.3. ATZ Ameliorates High-Fat Diet-Induced Impairment in Glucose Metabolism

**Figure 6:**
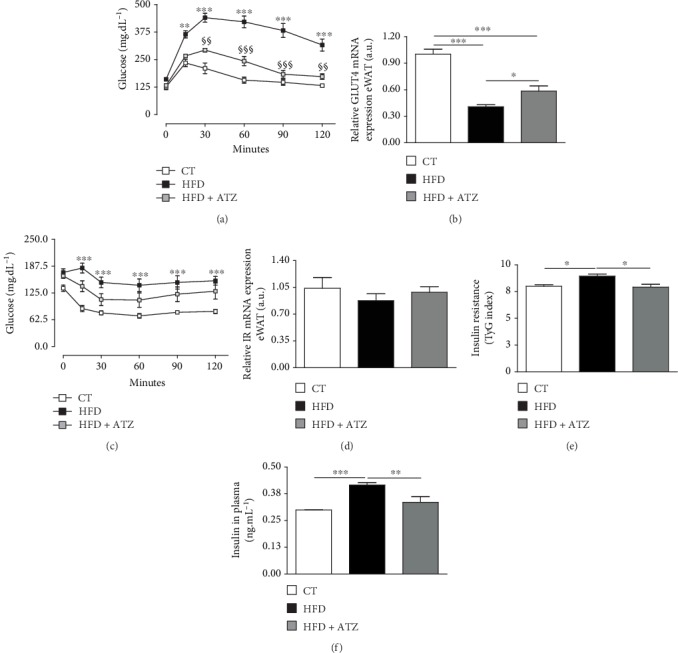
(a) Blood glucose levels in the glucose tolerance test (mg·dL^−1^); (b) relative gene expression of GLUT4 in eWAT (arbitrary units); (c) blood glucose levels in the insulin tolerance test (mg·dL^−1^); (d) relative gene expression of insulin receptor (IR) in eWAT (arbitrary units); (e) insulin resistance (TyG index); (f) insulin levels in plasma (ng·mL^−1^) of C57BL/6 mice after dietetic intervention (chow and high-fat) and oral treatment with ATZ (500 mg·kg^−1^). Each point on the graph represents the mean ± SEM. ANOVA (two-way): ^∗∗^*p* < 0.01; ^∗∗∗^*p* < 0.001 HFD *vs.* CT; ^§§^*p* < 0.01; ^§§§^*p* < 0.001 HFD+ATZ *vs.* HFD. Each bar graph represents the mean ± SEM. ANOVA (one-way): ^∗^*p* < 0.05, ^∗∗^*p* < 0.01, and ^∗∗∗^*p* < 0.001.

Fasting blood glucose was clearly increased in both groups fed with HFD (Table 2). Glucose tolerance was impaired after HFD consumption but not when the animals were treated with ATZ (Figure 6(a)); this is observed by the significant increase in the glycemic curve over time in the HFD group compared to the CT group, as well as the significant decrease in the same curve in the HFD+ATZ group compared to the HFD group, evidencing the opposite effect of HFD and ATZ on glucose tolerance. The mRNA for GLUT4 in eWAT was significantly decreased in the HFD group compared to the CT group, and ATZ attenuated this effect (Figure 6(b)). Accordingly, the increase in insulin resistance observed by the significant increase in the glycemic curve over time in HFD animals was also attenuated in the ATZ-treated group, although not significantly compared to the HFD group (Figure 6(c)). The gene expression of insulin receptor in eWAT was not different between the groups (Figure 6(d)). Nevertheless, the TyG index and plasmatic insulin levels were significantly higher in the HFD than in the CT group, and ATZ significantly reduced these parameters (Figures 6(e) and 6(f)). Together, these data suggest that ATZ ameliorates HFD-induced impairment of glucose metabolism.

## 4. Discussion

Long-term oral treatment with ATZ provides strong reduction both in fat deposition and in body weight in HFD-induced obesity mice, accompanied by an improved lipid and glucose metabolism.

We consider two pathways by which ATZ could induce the strong fat loss: in a first line of reasoning, the catalase inhibition and, consequently, the increase in H_2_O_2_ levels, or in a second line of reasoning, the blocking of heme synthesis by inhibiting aminolevulinic acid dehydratase.

The role of catalase in adiposity is still controversial. On the one hand, catalase overexpression in fat inhibits adiposity [29]. On the other hand, knockout animals for catalase show different metabolic profiles according to the literature. The work of Park et al. [9] shows no effect of catalase knockout on body weight and epididymal fat mass either under normal diet or HFD conditions compared to wild type. However, plasma-free fatty acids and triglycerides were increased in catalase knockout animals [9]. Heit et al. [30], in turn, showed that catalase knockout animals presented an increase in body weight and serum triglycerides, despite no difference in WAT weight between wild-type and knockout mice. Our findings identify the opposite after ATZ treatment: the animals decreased all parameters related to HFD-induced obesity, although catalase activity was not completely inhibited in WAT, which supported our second line of reasoning for the mechanism of action for ATZ: heme synthesis inhibition which, in turn, blocks adipogenesis.

ATZ is an inhibitor of heme biosynthesis [31], which, in turn, leads to blockage of adipogenesis probably by activation of nuclear receptor RevErb*α* [14, 32]. Under HFD, this process leads to apoptosis of adipocytes, followed by the invasion of inflammatory cells. The increase in NEFA release, as evidenced by lipolysis *in vivo*, a marked atrophy in WAT, and the decrease in adipocyte diameter are consequences of blocking adipogenesis, enhanced inflammation, and apoptosis induced by ATZ. Also, most of our gene expression data for inflammatory and cell death markers fit to this scenario: increase in CD68, CCL2, caspase 1, TNF-*α*, IL-1*β*, IL-18, and CIDEA in WAT. In addition, the WAT of the ATZ group presented a high amount of macrophages. During adipose tissue inflammatory response, such as that occurring in obese WAT, CCL2 binds its receptor CCR2 activating the monocyte transmigration and differentiation into M1 macrophages. The classical M1 pathway induces the production of proinflammatory cytokines (for example, IL-1*β*, IL-6, and TNF-*α*) [33, 34], which concur with the scenario observed in WAT of the ATZ-treated group. Corroborating the findings of our data, Park et al. [9], who used knockout mice for catalase, as well as mice that received ATZ as a pharmacological inhibitor of catalase, showed macrophage infiltration into WAT [9].

Functioning as a RevErb*α* ligand, heme is an important signaling molecule for the induction of adipogenesis [35]. Our hypothesis is that ATZ may interfere with the adipogenic programming probably by activation of nuclear receptor RevErb*α*, a pivotal player in adipocyte differentiation [14, 35]. Fontaine et al. [36] identified the gene of RevErb*α* as a target for nuclear receptor PPAR*γ* [37–39] in adipogenesis and as a modulator of adipocyte function. PPAR*γ* hypomorphic mice (*PPARγ^hyp/hyp^*) presented neonatal mortality; the surviving animals exhibited a lipodystrophy, with moderate glucose intolerance but not a fatty liver, and compensatory regulation of genes in the muscle that allowed the oxidation of lipid excess [40]. On the other hand, adipose-specific PPAR*γ* knockout causes insulin resistance in the fat and liver, and these animals are more susceptible to HFD-induced insulin resistance and steatosis [37]. Together, these studies highlight the relevance of PPAR*γ* in lipid and glycemic metabolism, as well as support the idea that the observed improvement of these pathways in obese animals treated with ATZ may be a reflection of the high level of PPAR*γ* associated with fat loss.

Wang and Lazar [14] identified a bifunctional role of RevErb*α* in adipocyte differentiation in vitro. Their work showed that during adipogenesis, the mRNA increases but protein expression behaves differently: during the initial stages of differentiation it increases, because of being required for the early mitogenic event during 3T3-L1 cell adipogenesis. Still, at later stages, RevErb*α* protein levels are decreased, because its degradation is necessary for continued differentiation, since it inhibits the adipogenic programming by repressing the expression of PPAR*γ*. According to these data, we would expect an increase in the gene expression of RevErb*α* and a decrease in PPAR*γ*, but both were increased in the ATZ group, suggesting that the augmentation of PPAR*γ* is compensatory to the drastic loss of fat. Accordingly, data from our group showed that mice treated for 5 days with ATZ exhibited decreased PPAR*γ* mRNA in WAT (data not shown), confirming that PPAR*γ* expression is indeed suppressed in the beginning of treatment.

As a result of fat loss and probably also the increase in PPAR*γ*, previously discussed, the animals treated with ATZ showed improvement in several parameters that were impaired by consumption of HFD: the decrease in leptin plasma levels and gene expression in WAT, regulating energy intake and expenditure [41]; the increase in HSL gene expression, contributing to lipolysis [42]; the increased BAT amount, which may reflect on thermogenic improvement [43]; the amelioration of insulin and glucose regulation evidenced by the decrease in glucose intolerance, insulin resistance, and insulin plasma levels, as well as the increase in GLUT4 expression in WAT, the main glucose transporter expressed in adipose tissue [44]; and the decrease in plasma lipid levels. Moreover, the ATZ treatment decreased the liver weight, which may reflect the decrease in hepatic lipid deposition (HFD = 0.0960 ± 0.01 vs. HFD + ATZ = 0.0695 ± 0.01; *p* < 0.05). ATZ decreased the adiponectin mRNA in WAT; we consider two alternatives to explain: in a first line of reasoning, it could be attributed to the drastic decrease in the WAT index, or in a second line of reasoning, the blocking of adipogenesis, which also interferes with the production of anti-inflammatory cytokines such as adiponectin. In addition, the increase in catalase and the decrease in SOD gene expression in WAT could indicate a compensatory mechanism related to the partial inhibition of catalase. Interestingly, chronic ATZ-treatment diminished HFD-induced lipid peroxidation, a marker for oxidative stress [5].

The heme hypothesis is not consistent with all data. It is possible that ATZ performed a mixed action in adipose tissue. Thus, we cannot discard other ATZ effects, such as inhibition of acetyl-CoA carboxylase and thereby fatty acid synthesis in the liver [45], which can contribute to the decrease in lipid depots. Nevertheless, the exact mechanism involved in the ATZ action in body weight and WAT depots, as well as in redox signaling, be it local or systemic, needs more investigations.

**Figure 7:**
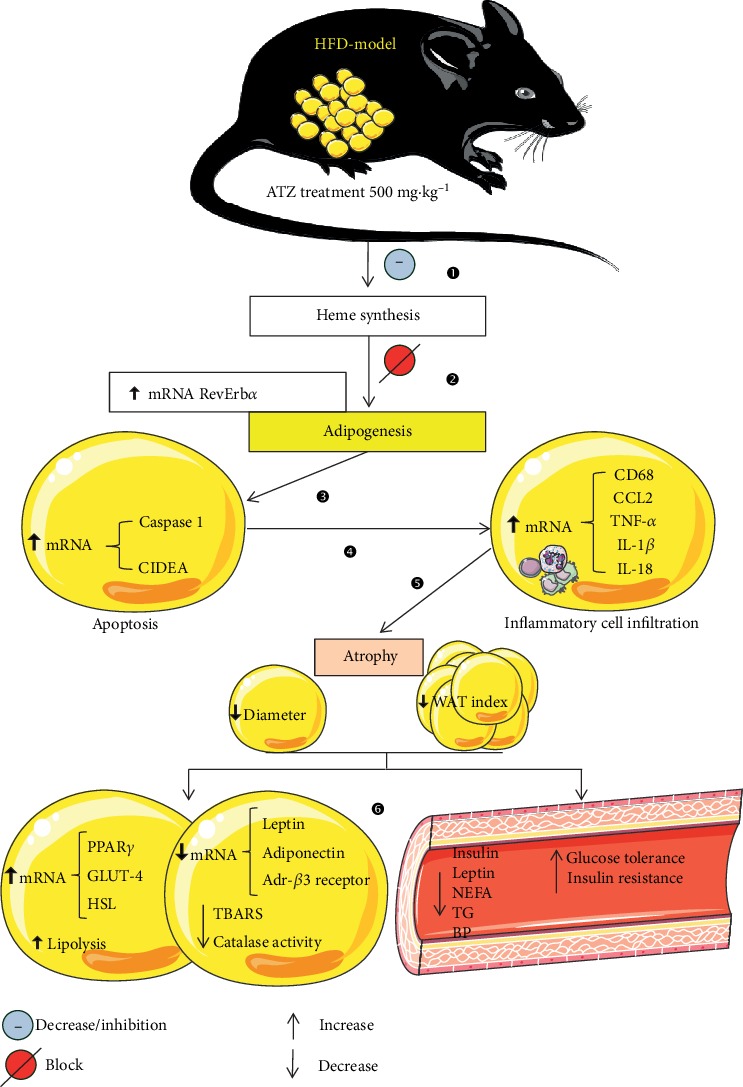
Proposed mechanism by which ATZ acts: in the context of HFD-induced obesity and metabolic syndrome in mice (HFD-model), ATZ treatment probably induces heme synthesis inhibition/decrease (1), which blocks adipogenesis by increasing RevErb*α* gene expression and, possibly, decreasing RevErb*α* activity (2), leading to apoptosis of adipocytes, evidenced by the increases in mRNA for caspase 1 and cell death activator (CIDEA) (3). This condition promotes inflammatory cell infiltration in epididymal white adipose tissue (eWAT), confirmed by the increased expression of marker genes: cluster of differentiation 68 (CD68), monocyte chemoattractant protein 1 (MCP1 or CCL2), tumor necrosis factor alpha (TNF-*α*), interleukin 1*β* (IL-1*β*), and interleukin 18 (IL-18) (4), inducing atrophy in eWAT, as well as the decreases in adipocyte diameter and WAT index (5). As a consequence of fat loss, ATZ elicits a beneficial systemic antiobesity effect and improves the metabolic status by increasing lipolysis *in vivo*, peroxisome proliferator-activated receptor gamma (PPAR*γ*), glucose transporter 4 (GLUT4), and hormone-sensitive lipase (HSL) genes, as well as by decreasing lipid peroxidation (TBARS), catalase activity, and mRNA for leptin, adiponectin, and beta-3 adrenergic receptor (Adr-*β*3 receptor) in eWAT. In addition, systemically, ATZ decreases insulin, leptin, nonesterified fatty acid (NEFA), triglyceride (TG) levels, and blood pressure, which results in the improvement of glucose tolerance and insulin sensitivity (6).

Collectively, we show that ATZ induces quick and strong fat loss probably through heme synthesis inhibition which blocks adipogenesis by probably decreasing RevErb*α* activity. As a consequence, adipocytes become apoptotic, which leads to the recruitment of macrophages and inflammation in WAT. This strong antiobesity effect of ATZ together with the low toxicity of this compound [46], and the beneficial secondary effects on blood pressure, lipid and glucose metabolism, could make it a new treatment option for both obesity and MetS. According to the results of the present work, Figure 7 highlights the proposed mechanism by which ATZ acts.

## 5. Summary

Our findings give new insight into the action of ATZ on lipid metabolism. Considering its low toxicity, ATZ could represent an old compound with novel mechanisms of action to be studied for the treatment of both obesity and metabolic syndrome.

## Figures and Tables

**Table 1:** Primer sequences used for real-time quantitative PCR (amplicons between 100 and 150 bp).

Primers	Sequence forward and reverse (5′–3′)
Adiponectin	F: GGAACTTGTGCAGGTTGGAT
	R: CCTTCAGCTCCTGTCATTCC
Adr*β*3	F: GCTGACTTGGTAGTGGGACTC
	R: TAGAAGGAGACGGAGGAGGAG
Caspase 1	F: ACCCTCAAGTTTTGCCCTTT
	R: GATCCTCCAGCAGCAACTTC
Catalase	F: CACGCTGGTAGTTGGCCACT
	R: GCCCAGCCCTGACAAAATGC
CCL2	F: GCCAACTCTCACTGAAGCC
	R: GCTGGTGAATGAGTAGCAGC
CD68	F: TAGGACCGCTTATAGCCCAAG
	R: CTGTAGGTGTCATCGTGAAG
CIDEA	F: GCAGCCTGCAGGAACTTATC
	R: CCGATTTCTTTGGTTGCTTG
GAPDH	F: CCATCACCATCTTCCAGGAG
	R: GTGGTTCACACCCATCACAA
GLUT4	F: TGATTCTGCTGCCCTTCTGT
	R: GGACATTGGACGCTCTCTCT
HSL	F: ACGGATACCGTAGTTTGGTGC
	R: TCCAGAAGTGCACATCCAGGT
IR	F: CCACCAATACGTCATTCACAAC
	R: GGGCAGATGTCACAGAATCAA
Interleukin-1*β*	F: GCCACCTTTTGACAGTGATGAG
	R: CCTGAAGCTCTTGTTGATGTGC
Interleukin 18	F: TCTGACATGGCAGCCATTGT
	R: CAGGCCTGACATCTTCTGCAA
Lep	F: CGTGTGTGAAATGTCATTGATCCT
	R: GACACCAAAACCCTCATCAAGAC
LPL	F: AGTGGCCGAGAGCGAGAAC
	R: CCACCTCCGTGTAAATCAAGAAG
PPAR*γ*	F: TCAGCTCTGTGGACCTCTCC
	R: ACCCTTGCATCCTTCACAAG
RevErb*α*	F: TGGCCTCAGGCTTCCACTATG
	R: CCGTTGCTTCTCTCTCTTGGG
SOD1	F: GACGGTGTGGCCAATGTGTC
	R: CAAGCGGCTCCCAGCATTTC
TNF-*α*	F: GTCTACTGAACTTCGGGGTGA
	R: CTCCTCCACTTGGTGGTTTG
UCP2	F: GCATTGGCCTCTACGACTCT
	R: GTCCTGGTATCTCCGACCAC

Adr*β*3: adrenergic *β*3 receptor; CCL2 or MCP1: monocyte chemoattractant protein 1; CD68: cluster of differentiation 68; CIDEA: cell death activator; GAPDH: glyceraldehyde 3-phosphate dehydrogenase; GLUT4: glucose transporter type 4; HSL: hormone-sensitive lipase; IR: insulin receptor; Lep: leptin; LPL: lipoprotein lipase; PPAR*γ*: peroxisome proliferator-activated receptor gamma; RevErb*α* or NR1D1: nuclear receptor subfamily 1; SOD1: superoxide dismutase 1; TNF-*α*: tumor necrosis factor alpha; UCP2: uncoupling protein 2.

**Table 2:** General characteristics from control, HFD, and HFD+ATZ (500 mg·kg^−1^) treated mice.

Parameter	Groups		
	CT	HFD	HFD+ATZ
Final systemic fasting glucose	103.3 ± 7.1	180.2 ± 13.86^a^	154.5 ± 5.65^c^
Food intake (g/body weight·day^−1^)	0.16 ± 0.00	0.081 ± 0.00^a^	0.08 ± 0.00
Final body weight (g)	32.19 ± 0.27	47.87 ± 1.41^a^	27.83 ± 0.20^b,c^
*Δ* body weight (final minus basal)	10.95 ± 0.51	25.69 ± 0.89^a^	7.99 ± 0.59^b,c^
*Δ* body weight (g) (final minus start of ATZ treatment)	3.78 ± 0.63	7.40 ± 0.77^a^	−7.31 ± 0.90^b,c^
Systolic blood pressure	118.0 ± 2.44	134.8 ± 5.16^a^	118.9 ± 3.27^b^
Epididymal adipose tissue (%)	1.36 ± 0.13	3.05 ± 0.19^a^	0.26 ± 0.06^b,c^
Perirenal adipose tissue (%)	0.67 ± 0.18	1.67 ± 0.22^a^	0.08 ± 0.02^b,c^
Brown adipose tissue (%)	0.32 ± 0.03	0.33 ± 0.01	0.84 ± 0.04^b,c^
Total cholesterol (mg·dL^−1^)	85.78 ± 2.84	135.6 ± 10.59^a^	110.3 ± 20.26
Triglycerides (mg·dL^−1^)	57.70 ± 8.6	83.98 ± 15.04	41.51 ± 8.7^b^
Nonesterified fatty acid (NEFA-mmol·L^−1^)	0.38 ± 0.04	0.60 ± 0.05^a^	0.17 ± 0.0^b,c^
Right tibia (mm)	18.1 ± 0.01	17.8 ± 0.03	18.4 ± 0.02
Total plasma proteins (mg·mL^−1^)	7.96 ± 0.07	8.13 ± 0.14	8.01 ± 0.05

Values represent the mean ± SEM. ANOVA one-way: ^a^*p* < 0.05 HFD vs. CT. ^b^*p* < 0.05 HFD+ATZ vs. HFD. ^c^*p* < 0.05 HFD+ATZ vs. CT.

## Data Availability

The data used to support the findings of this study are available from the corresponding author upon request.

## Abbreviations

Adr*β*3: Beta-3 adrenergic receptor
ATZ: 3-Amino-1,2,4-triazole
BAT: Brown adipose tissue
BIH: Berlin Institute of Health
CD68: Cluster of differentiation 68
CIDEA: Cell death activator
CT: Control
DZHK: German Center for Cardiovascular Research
eWAT: Epididymal white adipose tissue
FANUT: Faculdade de Nutrição
GLUT4: Glucose transporter 4
H_2_O_2_: Hydrogen peroxide
HFD: High-fat diet
HSL: Hormone-sensitive lipase
ICBS: Instituto de Ciências Biológicas e da Saúde
IL-1*β*: Interleukin 1*β*
IL-18: Interleukin 18
IR: Insulin receptor
LPL: Lipoprotein lipase
MCP1 or CCL2: Monocyte chemoattractant protein 1
MetS: Metabolic syndrome
NEFA: Nonesterified fatty acid
PPAR*γ*: Peroxisome proliferator-activated receptor gamma
RevErb*α* or NR1D1: Nuclear receptor subfamily 1
SOD: Superoxide dismutase
TBARS: Thiobarbituric acid reactive substances
TCOL: Total cholesterol
TG: Triglycerides
TNF-*α*: Tumor necrosis factor alpha
TyG index: Triglyceride and glucose levels index
UCP2: Uncoupling protein
UFAL: Universidade Federal de Alagoas
UFPE: Universidade Federal de Pernambuco
WAT: White adipose tissue.

## Additional Points

*Highlights*. 3-Amino-1,2,4-triazole (ATZ) is a potent antiobesity agent in HFD-induced metabolic syndrome mice. ATZ is a potent inducer of white fat loss and body weight reduction. ATZ may act through heme synthesis inhibition, which blocks adipogenesis by the increase in mRNA RevErb*α*. Under HFD, this ATZ action leads to apoptosis of adipocytes and the invasion of inflammatory cells. The drastic and rapid decrease in white adipose tissue induced by ATZ improved metabolic parameters.
